# Genetic Models of Leukemia in Zebrafish

**DOI:** 10.3389/fcell.2018.00115

**Published:** 2018-09-20

**Authors:** Jeremy T. Baeten, Jill L. O. de Jong

**Affiliations:** Department of Pediatrics, University of Chicago, Chicago, IL, United States

**Keywords:** zebrafish, leukemia, animal models, ALL, AML, MDS, MPN

## Abstract

The zebrafish animal model is gaining increasing popularity as a tool for studying human disease. Over the past 15 years, many models of leukemia and other hematological malignancies have been developed in the zebrafish. These confer some significant advantages over similar models in other animals and systems, representing a powerful resource for investigation of the molecular basis of human leukemia. This review discusses the various zebrafish models of lymphoid and myeloid leukemia available, the major discoveries that have been made possible by them, and opportunities for future exploration.

## Introduction

### Leukemia

Leukemia is a broad designation encompassing hematological malignancies that produce the expansion of blood cells, typically starting in the bone marrow. In 2015, there were over 2.3 million patients suffering from leukemia, resulting in over 350,000 deaths worldwide (GBD 2015 Mortality and causes of death collaborators, 2016). In the United States, an estimated 62,130 new leukemia cases were diagnosed and 24,500 deaths caused by leukemia in 2017, with a 5 years survival rate of ∼63% (NCI SEER Cancer Stat Facts: Leukemia). Although the majority of leukemias affect adults, leukemia is also the most common cancer diagnosis in children.

Leukemias are categorized by two major criteria into four groups. The first criterion relates to the cell of origin: leukemias of lymphoid origin are classified “lymphocytic or lymphoblastic” and those of myeloid origin called “myelogenous or myeloid.” The second criterion deems rapidly growing leukemias as “acute” and those with more indolent growth as “chronic.” The causes of these different malignancies are varied. Some are directly linked to a chromosomal abnormality, such as the Philadelphia chromosome in chronic myelogenous leukemia (Bartram et al., 1983) or the increased incidence of leukemia in patients with trisomy 21 (down syndrome) (Evans and Steward, 1972). However, the etiology of most leukemias is less straightforward. Some leukemias involve mutations and/or translocations of multiple genes associated with growth, differentiation and survival of blood cells. Others have a normal karyotype and no known genetic mutations, highlighting the need for further studies in animal models to uncover these unknown drivers of hematopoietic malignancy.

### Zebrafish as a Model Organism

**FIGURE 1:**
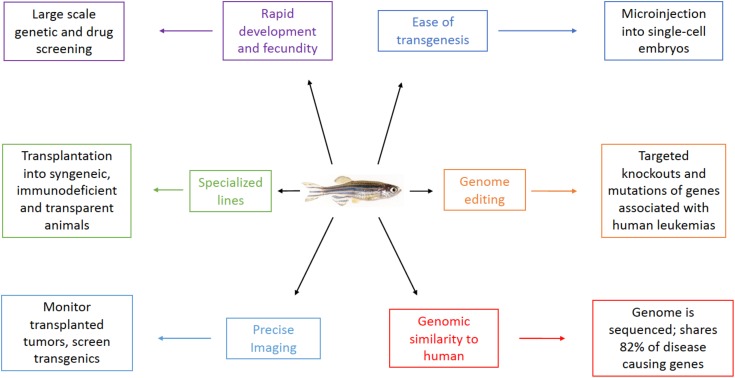
Advantages of the zebrafish model for leukemia research.

*Danio rerio*, commonly known as the zebrafish, is a small tropical fish popular in pet stores and aquariums. Since the 1970’s when George Streisinger first began using the zebrafish as a model organism (Walker and Streisinger, 1983), more and more labs have begun utilizing this powerful tool for studying development and disease due to numerous advantages over other model systems (**Figure 1**).

Zebrafish fertilization and development occurs externally in optically clear embryos that are easily observed and manipulated. Development is much faster than mammals, with most major organs forming by 2–3 days post-fertilization (dpf). Animals reach sexual maturity by 2–3 months of age (Kimmel et al., 1995) and a single breeding pair produces several hundred embryos weekly. This fecundity coupled with their rapid development makes the zebrafish an excellent model for large-scale screening. Forward and reverse genetic screens as well as toxicity and drug screens in zebrafish have been performed around the world over the past three decades (Ransom et al., 1996; Weinberg et al., 1996; Sood et al., 2006; North et al., 2007; Ridges et al., 2012), including significant work more recently to evaluate therapeutics in zebrafish leukemia models (Mizgirev and Revskoy, 2010; Deveau et al., 2017). Although teleosts (like the zebrafish) and mammals diverged from a common ancestor approximately 340 million years ago, they still share a remarkable amount of their genomes, with a zebrafish ortholog identified for 82% of known disease-causing genes in humans (Howe et al., 2013). Many of these zebrafish genes have already been shown to recapitulate human disease when affected in zebrafish, including several connected to hematopoiesis (Brownlie et al., 1998; Wang et al., 1998) and cancer, as we will discuss in this review.

Several systems have been developed within the zebrafish model to create transgenic and knockout animals. Because the zebrafish embryos are externally developed, it is possible to microinject directly into the single-cell for the first 15–30 min following fertilization. Although, the first transgenic zebrafish were created through injection of naked, linearized DNA (Stuart et al., 1988), more efficient systems of genomic incorporation are now available. The tol2 transposon system creates randomly inserted transgenes that heavily favor single copy insertions (Urasaki et al., 2006) and the I-SceI meganuclease system inserts one or more copies into double-stranded breaks in the genome (Grabher et al., 2004; Ogino et al., 2006). In addition to the ability to integrate transgenes, the advent of CRISPR/Cas9 technology has made it possible to directly edit the zebrafish genome; from creating knockouts to mimicking human mutations to introducing specific SNPs. The use of CRISPR/Cas9 in zebrafish was first described by Keith Joung’s lab in 2013 (Hwang et al., 2013), and has since spread throughout the field to become a common tool in many labs’ arsenal, just as it has throughout the biomedical community at large (Hruscha et al., 2013; Ablain et al., 2015).

Over the years, the zebrafish community has amassed a large number of inbred, transgenic, knockout, or other specialized lines that have been characterized and maintained for various applications. Important to leukemia models are several lines that allow for transplantation of tumors without the need for pre-transplant immune ablation. Generated by parthenogenesis, the clonal golden lines (CG1 and CG2) allow for syngeneic transplantation within a genetically identical line, similar to transplantation experiments using inbred mice (Mizgireuv and Revskoy, 2006; Smith et al., 2010). The *rag2* (E450fs) mutant line has reduced numbers of functional T- and B-cells, and thus is unable to mount a significant immune response against transplanted cells (Tang et al., 2014). The *c-myb*^I181N^ hypomorphic mutant is another immunocompromised line that has shown promise in xenograft experiments (Hess and Boehm, 2016). These lines allow for immunologically unmatched transplantation from other zebrafish lines as well as xenografts.

A common challenge for many model systems is the ability to visualize and trace the fate of a cancer cell within an animal over time. These issues are often circumvented by euthanizing, sectioning, and staining multiple animals at different timepoints, however, this increases the number of animals required, increases time commitment, and may blur inter-individual variability. In the zebrafish, fluorescently tagged proteins or cells can be clearly imaged from embryo to adulthood in live animals by confocal or lightsheet microscopy (Kaufmann et al., 2012) and at even greater resolutions in the pigment-less Casper line (White et al., 2008). An excellent example of this utility was described by Kaufman et al. (2016), when they used a *crestin-EGFP* line to show melanoma initiation and progression from a single cell. Also, with the macroscope developed by the Langeneau lab, high-throughput imaging of adult fish is possible for transgenic lines or screening for tumor engraftment in transplantation models (Blackburn et al., 2011).

### Hematopoiesis: Zebrafish and Human

Many of the transcription factors and major signaling pathways controlling hematopoietic differentiation are mutated or dysregulated in the transformation and progression of leukemia. Therefore, in addition to the general advantages described above, the zebrafish is an appealing model for studying leukemia because of the close parallels to mammalian hematopoiesis (de Jong and Zon, 2005). Though the locations of hematopoiesis are not perfectly shared between species, the ontogeny of the different hematopoietic cells from progenitors to maturity, as well as the genes and pathways driving differentiation are well conserved (Paik and Zon, 2010). There are two distinct waves of hematopoiesis in all vertebrates; a transient primitive wave supplying necessary macrophages and erythrocytes for early embryonic development, followed by the definitive wave that gives rise to the full complement of blood cells throughout an animal’s lifetime. In mammalian development, the primitive hematopoietic stem cells (HSCs) appear within the blood islands in the embryonic yolk sac (Palis and Yoder, 2001). In zebrafish, these limited HSCs instead arise from the intermediate cell mass (ICM) within the ventral mesoderm, and, similarly, produce erythrocytes and other myeloid cells (Detrich et al., 1995). Expression of the transcription factors *scl*, *gata2*, *lmo2*, *tif1γ*, and *fli1* promotes the primitive HSC lineage (Liao et al., 1998; Thompson et al., 1998; Ransom et al., 2004), while *gata1* and *spi1* (also known as *pu.1)* drive their differentiation into the erythroid and myeloid lineages, respectively (Detrich et al., 1995; Lieschke et al., 2002).

The mammalian definitive wave of hematopoiesis begins with true multipotent HSCs emerging from the ventral wall of the dorsal aorta in the aorta-gonad-mesenephros (AGM) region that then migrate to the fetal liver to proliferate and differentiate, and ultimately migrate to seed the bone marrow (Cumano and Godin, 2007). This process is mirrored in the zebrafish with the definitive HSCs also arising from the ventral wall of the dorsal aorta, and migrating to the caudal hematopoietic tissue (CHT) before seeding the kidney marrow, which is the zebrafish adult hematopoietic tissue (Burns et al., 2002; Jin et al., 2007). Definitive HSCs are true multipotent hematopoietic progenitors and are marked by their expression of the transcription factors *runx1*, *c-myb*, *lmo-2*, and *scl* (Thompson et al., 1998; Burns et al., 2002). Similar to primitive hematopoiesis, *gata1* and *tif1γ* drive erythropoiesis (Detrich et al., 1995; Ransom et al., 2004) and *spi1* and *c/ebp1* drive myelopoiesis (Lyons et al., 2001; Lieschke et al., 2002). Unlike the primitive lineages, definitive HSCs also produce lymphoid cells through expression of *rag1*, *rag2*, *ikaros*, *lck*, and *gata3* (Willett et al., 1997, 2001; Langenau et al., 2004). There are several functional and structural differences in the hematopoietic system of zebrafish compared to mammals, namely the location of the marrow, the lack of lymph nodes, and the rapid development and early reliance on the innate immune system (Novoa and Figueras, 2012; Renshaw and Trede, 2012). However, ultimately the blood cells of the zebrafish and human are molecularly very similar and thus have common genetic drivers of leukemia.

The conservation of the genes and pathways regulating hematopoiesis between humans and zebrafish, combined with the significant technical advantages provided by this model animal, make the zebrafish an ideal system for investigating hematological malignancies. In this review, we will examine the many leukemia models that have been developed within the zebrafish, and discuss the major findings made possible by each model that have advanced our understanding of human leukemia.

## Zebrafish Leukemia Models: Lymphoid Origin

The first leukemia model in zebrafish was developed over 15 years ago using the lymphocyte-specific *rag2* promoter driving the murine *c-Myc* oncogene to produce T-cell acute lymphoblastic leukemia (T-ALL) (Langenau et al., 2003). The success of that first step has spawned a variety of other models tied to different types of leukemia. Over time, these models have been altered and improved to fit the particular investigations of each project, and there are now multiple similar models available, each with their own strengths and weaknesses (**Table 1**). This section discusses models of lymphoid origin, and the major discoveries made possible by them.

**Table 1:** Zebrafish leukemia models of lymphoid origin.

Model	Gene/pathway; expression	Model features	Major findings
**T-ALL**			
*rag2: EGFP-mMyc*	Murine *c-Myc*oncogene (*mMyc*);thymus	Stable transgenesis of GFP-tagged *Myc*, line must be propagated by IVF	First leukemia model in zebrafish, similar disease progression to human T-ALL (Langenau et al., 2003)
*Microinjected rag2: EGFP-mMyc*		GFP-tagged, microinjected into single-cell embryos	Non-IR transplantation in CG1 line (Smith et al., 2010); undergo clonal evolution, AKT activation increases LSCs and resistance (Blackburn et al., 2014); subset of B-ALL and bi-phenotypic tumors (Garcia et al., 2018)
*rag2: Myc-ER;*	Tamoxifen-inducible *mMyc*; thymus	4OHT treatment after 5 dpf, induction at ∼35 dpf, not fluorescently labeled	Loss of *Myc* leads to apoptosis, PTEN/AKT-MYC axis (Gutierrez et al., 2011); loss of *bim* promotes *Myc*-independent T-ALL survival (Reynolds et al., 2014)
*rag2: loxP-dsRed2-loxP-EGFP-mMyc*	Cre-inducible *mMyc*; thymus	Cre-induces *mMyc* transformation and red to green color change; 81% efficient with *hsp70:Cre* and heat shock (Feng et al., 2007)	Progression similar to *rag2: EGFP-mMyc* (Langenau et al., 2005); with *rag2-EGFP-bcl-2*: accelerates T-LBL and autophagy, inhibits T-ALL progression and intravasation (Feng et al., 2010)
*rag2: EGFP-ICN1*	*notch1* intracellular domain; thymus	High latency (∼11 months), GFP-tagged	Increased expression of Notch targets *her6/9*; cooperation with *rag2-EGFP-bcl-2* increases onset/incidence, survival and resistance to irradiation (Chen et al., 2007); enhances T-ALL progression in combination *with rag2:cMyc*, does not increase LSC frequency, molecularly similar to human disease (Blackburn et al., 2012)
*Srk* *Hlk* *Otg*	ENU mediatedmutagenesis in*Lck:EGFP lines;*thymus	Genes affected not reported; high latency (5–10 months to incidence)	Establishes viability of mutagenesis screen, serially transplanted tumors are increasingly malignant (Frazer et al., 2009)
**B-ALL**			
*β-actin: EGFP-TEL-AML1* *xEf1α:EGFP-TEL-AML1*	Human *TEL-AML1*(ETV6-RUNX1)fusion;global	Low incidence (3%), long latency (8–12 months); similar to CD10+ preB**-**ALL	Only zebrafish model of B**-**ALL; Likely requires secondary mutation; deregulation of survival genes; *rag2-* driven *TEL-AML* does not produce B**-**ALL, needs early precursor expression (Sabaawy et al., 2006)

### T-cell Acute Lymphoblastic Leukemia (T-ALL)

The majority of lymphoid leukemia models in zebrafish replicate T-ALL, partially due to the success of the *rag2* promoter in driving that particular malignancy. Although *rag2* is expressed in both T- and B-cell precursors in zebrafish (Langenau et al., 2004), only T-cell leukemias were initially identified from models utilizing this promoter. Interestingly, the Langenau lab has recently published a brief communication describing a subset of B-cell derived and bi-phenotypic leukemias produced from a rag2 promoter (Garcia et al., 2018), suggesting some of the research done on these T-ALL models may have unknown contributions from B-ALL as well.

The oncogene *c-Myc* is associated with many cancers and is one of the most frequently affected gene pathways in lymphoid leukemia (La Starza et al., 2014). The first T-ALL model was developed by Langenau et al. by expressing the murine *c-Myc* oncogene under the zebrafish *rag2* promoter, with an EGFP tag for easy monitoring by fluorescent microscopy (Langenau et al., 2003). Tumors were generated in microinjected mosaic F_0_ fish at similar rates to *EGFP* expression in control animals injected with *rag2*: *EGFP*, suggesting complete penetrance of tumor induction upon successful integration of the *c-Myc* transgene. These tumors grew rapidly, with a mean latency of 52 days post-fertilization (dpf), extensively infiltrating the entirety of the fish. Analysis of the expression profiles and T-cell receptor (TCR) rearrangements confirmed that the tumor cells derived from clonal expansion of transformed T lymphocyte precursors and originated in the thymus. Tumor cells could be transplanted into irradiated recipients and quickly grew new tumors that homed to the thymus before spreading throughout the animal. Overall, the tumors progressed, similarly, to human T-ALL, at an accelerated pace. However, most F1 progeny developed advanced disease well before reaching sexual maturity (mean latency 32 dpf), necessitating sperm collection and *in vitro* fertilization (IVF) to continue the stable transgenic line. Subsequent characterization of this model showed that the tumors express *tal1/scl* and *lmo2*, genes associated with a molecular subgroup of *Myc*-induced T-ALL in humans (Langenau et al., 2005).

To circumvent the necessity of IVF, Langenau et al. sought to create an inducible version of their model. They achieved this by inserting a loxP-DsRed2-loxP sequence cassette between the *rag2* promoter and *EGFP*-*mMyc* oncogene, creating the *rag2:loxP-dsRed2-loxP-EGFP-mMyc* line (*rag2: LDL-Emyc*) (Langenau et al., 2005). This allowed for default red fluorescent expression with a switch to *EGFP-mMyc* expression in the presence of Cre recombinase. The disease in these animals was morphologically similar to that in the *rag2:EGFP-mMyc* model, but with significantly decreased incidence (6.5%) and delayed latency (mean 151 dpf). This was presumed to be due to incomplete recombination of the transgene, as evidenced by the persistence of red fluorescent expression within the tumor. To combat this, they developed a heat shock-inducible Cre line, *hsp70: Cre* (Feng et al., 2007). When combined with their Cre-inducible *rag2: LDL-Emyc* line and subjected embryos to heat shock at 3 dpf, the penetrance (81%) and latency (120 dpf) were closer to those of the original *rag2: EGFP-mMyc* model. This improved model allowed them to explore the molecular events governing the progression of the disease from the localized T-lymphoblastic lymphoma (T-LBL) to disseminated T-ALL. All of the Myc-induced models of T-ALL in zebrafish begin as T-LBL with thymic hyperplasia and localized outgrowth before advancing to T-ALL and expanding into the circulation and other tissues. The investigation into this transition led them to combine the *rag2:LDL-Emyc; hsp70:Cre* model with a line overexpressing the survival gene *bcl2* (Feng et al., 2010). This combination accelerated T-LBL induction by suppressing Myc-induced apoptosis. However, it also promoted homotypic cell adhesion through *s1p1* and *icam1* that prevented intravasation into the vascular space and restricted the tumor to the thymus. The tumor cells then proliferated until they exhausted their nutrient supply and underwent autophagy. Because AKT-signaling is known to promote T-cell migration and to suppress autophagy (Sotsios and Ward, 2000; Lum et al., 2005), they hypothesized that addition of constitutively active AKT could force progression to T-ALL. Indeed, when their *Myc;Cre;bcl-2* embryos were injected with a myristolated-*akt2* transgene, the resulting tumors rapidly advanced to T-ALL (Feng et al., 2010).

The importance of AKT signaling in zebrafish T-ALL progression is not surprising, given the PTEN-PI3K-AKT pathway is frequently disrupted in human T-ALL (Palomero et al., 2008; Gutierrez et al., 2009). Gutierrez et al. further investigated this connection with the aid of another *Myc*-induced model, the tamoxifen inducible *rag2: Myc-ER* line (Gutierrez et al., 2011). This model allows for conditional expression of the *c-Myc* oncogene only in the presence of 4-hydroxytamoxifen (4-OHT). When continually treated with 4-OHT these fish develop T-ALL, but upon cessation of treatment and loss of *c-Myc* expression, the tumor cells undergo apoptosis and the tumor rapidly regresses. However, when AKT signaling was increased through either loss-of-function mutations in *pten* or constitutively active *akt2*, the tumors lost their dependence on *Myc* expression and were able to continue progression after removal of 4-OHT treatment. Further investigation into the relationship between *Myc* and the AKT pathway revealed that *Myc* drove the expression of the proapoptotic protein *bim*, while the constitutively active myr-*akt2* blocked that induction (Reynolds et al., 2014). Additionally, loss-of-function *bim* mutations allowed for increased persistence of T-ALL after cessation of 4-OHT treatment and *Myc* expression. Overall, these results suggest AKT-signaling enhances *Myc*-induced T-ALL progression via promotion of T-cell migration, suppression of autophagy, and inhibition of apoptosis.

Due to difficulty maintaining stable transgenic lines expressing *c-Myc*, an alternative approach was developed involving co-injection of the *rag2-EGFP* and *rag2-mMyc* transgenes into single-cell embryos (Langenau et al., 2008; Smith et al., 2010). In this model, the two transgenes randomly integrated into the genome to be co-expressed such that GFP expression was observed only in tumors and tumor induction only with GFP+ thymocytes. The resulting tumors followed the same pathology as the stable *Myc*-induced models. Smith et al. (2010) used this method to create tumors in clonal CG1 fish, demonstrating that they could be transplanted into syngeneic recipient CG1 fish without irradiation. This also allowed them to determine the frequency of leukemia stem cells (LSCs) present in these tumors through limit dilution analysis of the transplanted tumors. Each successful engraftment requires at least one LSC, and by transplanting different doses of cells, they were able to determine that 0.1–1.4% of the primary T-ALL tumor cells were LSCs. Transplantation of T-ALLs generated using this co-injection model was further investigated by Blackburn et al. (2014) who demonstrated that serial transplantation of T-ALL tumors led to spontaneous clonal evolution of monoclonal tumor subclones. As tumors were passaged from primary to secondary to tertiary recipients, some subclones evolved increased LSC frequency, growth, and/or resistance to therapy. Subclones with increased LSC frequency also displayed increased AKT phosphorylation, and treatment with an AKT inhibitor dramatically reduced their engraftment after transplant. Co-expression of myr-*akt2* with *Myc* significantly increased proliferation of tumor cells, decreased latency after transplantation, and increased LSC frequency sixfold, and these effects are at least partially due to AKT’s induction of *mtorc1* expression. Additionally, the subclones that had evolved glucocorticoid resistance were resensitized to dexamethasone treatment by AKT inhibitors. Altogether, these results provided further evidence of the connection between *Myc* and AKT in T-ALL.

Another major player in the transformation of T-cell precursors to T-ALL is *Notch1*, which has activating mutations in over 65% of T-ALL patients (Weng et al., 2004). To further study the role of *Notch1* in T-ALL, Chen et al. (2007) created a transgenic line expressing *rag2:ICN1-EGFP*, a GFP-tagged *Notch1* intracellular domain which acts as a constitutively active transcription factor to drive Notch target gene expression. This line develops T-ALL, but at a lower incidence (40%) and higher latency (>11 months) than the *Myc*-driven tumors. However, in the presence of bcl2 overexpression, the incidence (60–80%) and latency (40 dpf to induction; 3 months to dissemination) were significantly enhanced and apoptosis was decreased. Blackburn et al. further demonstrated this by combining the *rag2:ICN1-EGFP* and *rag2:cMyc* models which accelerated leukemia onset and incidence (Blackburn et al., 2012). They concluded that Notch signaling expanded pre-leukemic clones that required Myc (or acquired secondary mutations) to transform, and that Notch signaling did not increase the overall frequency of LSCs. They also used this model to make cross-species microarray comparisons with mouse and human T-ALL to identify a common T-ALL gene signature and novel Notch gene expression profile present in humans that is regulated independently of Myc. These two studies suggest that *Notch1* activation alone is not sufficient for induction of T-ALL and requires additional oncogene activation and/or tumor suppressor mutations.

Taking advantage of the ability to perform large-scale forward-genetic screens in zebrafish to identify genetic modifiers of disease, Frazer et al. (2009) developed one such screen for causative mutations in T-ALL using ENU-mediated mutagenesis of an *lck-EGFP* line. This screen identified three mutant lines that developed outgrowth of the GFP-tagged thymus and subsequently T-ALL. Two of these lines, *shrek (srk)* and *hulk (hlk)*, contained dominant mutations and one dubbed *Oscar-the-grouch (otg)* contained a recessive mutation. Homozygous fish from all 3 lines had incidences around 50% and time to tumor induction between 6 and 8 months. The mutated genes in these lines have not yet been reported, but the screen demonstrates the potential for identification of genes driving different leukemias. The lab also developed a chemical screen to identify small molecules capable of eradicating immature T-cells, using the same *lck-EGFP* line (Ridges et al., 2012). They identified Lenaldekar (LDK; 1H-indole-3-carbaldehyde 8-quinolinylhydrazone) as a compound capable of killing both normal and T-ALL blasts in zebrafish, and showed it was effective in mouse xenograft and human primary leukemia cells as well.

### B-Cell Acute Lymphoblastic Leukemia (B-ALL)

The *TEL-AML1* (also known as *ETV6-RUNX1*) fusion protein results from t(12;21), the most common translocation in childhood cancer, present in ∼25% of B-cell acute lymphoblastic leukemia (B-ALL) (Romana et al., 1995). However, attempts to produce a model of B-ALL from this fusion gene were unsuccessful in mice (Andreasson et al., 2001). Sabaawy et al. (2006) created multiple lines expressing human *TEL-AML1* from different promoters in zebrafish and were able to produce the only zebrafish model of B-ALL. Three different promoters were tested: the Xenopus *ef1a (Xef1a)* and zebrafish *beta-actin (zba)* for global expression, and zebrafish *rag2* for lymphocyte specific expression. Both of the global promoters produced B-ALL tumors in ∼3% of fish with 8–12 months latency and similar molecular and morphological features to pediatric CD10+ B-ALL. The low incidence likely indicates the need for a secondary mutation for oncogenic transformation. They surmised that the *rag2: TEL-AML1* fish did not develop tumors because the transformation occurs prior to the expression of Rag2 in the common lymphoid progenitor, and instead occurs in an earlier multipotent progenitor or hematopoietic stem cell in the global promoter lines. With the apparent T-cell bias of the *rag2* promoter in zebrafish, it also seems possible that a different promoter of common lymphoid or B-cell progenitors may have more success. However, the recent discovery of B-ALL in the rag2: cMyc fish provides an opportunity for studying B-ALL in a more accessible model, with much shorter latency and higher incidence (Garcia et al., 2018).

## Zebrafish Leukemia Models: Myeloid Origin

Following the initial success of the zebrafish ALL models, serious efforts began to recapitulate myeloid leukemias including myeloproliferative neoplasms (MPN) and acute myeloid leukemia (AML) in zebrafish. This was done largely through creating transgenic lines that expressed oncogenic fusion genes and mutations commonly found in patients with MPN and AML. This section discusses the features and major findings of the myeloid leukemia models developed to date in zebrafish (**Table 2**).

**Table 2:** Zebrafish leukemia models of myeloid origin.

Model	Gene/pathway; expression	Model features	Major findings
**AML and MPN**			
*spi-1: MYST3/NCOA2-EGFP*	Human *MYST3/NCOA2 (MOZ/TIF2)* fusion; Myeloid	EGFP-tagged, low incidence (1%) and high latency (14–26 months) in F_0_ fish	First AML model in zebrafish (Zhuravleva et al., 2008)
*spi-1: LGL-NUP98-HOXA9; hsp70-Cre*	Human *NUP98-HOXA9* fusion; Myeloid	Cre-conditional EGFP or transgene expression. Incidence ∼25%, latency 19–23 months	MPN-like disease, decreased apoptosis and cell cycle arrest in response to irradiation through bcl2 (Forrester et al., 2011); increased HSCs, oncogenesis requires *dnmt1* or *meis1*, epigenetic therapies restore normal hematopoiesis (Deveau et al., 2015)
*hsp70: AML1-ETO*	Heat shock-inducible human *AML-ETO* fusion; global	Embryonic loss of circulating blood cells, disrupted definitive hematopoiesis	Transcriptional changes mirror human AML, blocks *gata1* to bias granulocytes over erythrocytes (Yeh et al., 2008); embryonic screen of AML-therapeutics, COX and β-catenin are novel hematopoietic regulators/therapeutic targets (Yeh et al., 2009)
*CMV/Spi-1: tel-jak2a*	Zebrafish *tel-jak2a* mimicking human fusion; Global and myeloid	Embryonic Leukocyte expansion	ALL- and CML-derived fusions bias toward lymphoid or myeloid, respectively (Onnebo et al., 2012)
*β-actin: LGL-KRAS^G12D^; hsp70-Cre*	Cre-inducible Human *KRAS^G12D^* mutant; global	Multiple different malignancies; MPN incidence higher in non-heat shocked (53%), latency 66 dpf	MPNs are not transplantable past primary, does not confer self-renewal potential to progenitors. MPN can be induced by heat-shock *ex-vivo* (Le et al., 2007)
*HSE-MYCN-EGFP*	Heat shock inducible Murine *n-Myc*; Global expression	∼75% incidence in F_2_ fish, rapid onset (60 dpf), expanded myeloid populations in kidney/spleen	*n-Myc* can promote AML phenotypes, alters hematopoietic transcription factor expression (scl, lmo2, gata1, pu.1, runx1, cmyb) (Shen et al., 2013)
*spi1: FLT3-ITD-2A-EGFP*	Human *FLT3-ITD* mutant; Myeloid	Myeloid hyperplasia (6 months), AML-like (9 months)	Double mutants develop leukemia by 6 months (Lu et al., 2016)
*spi1*: *NPM1*-*Mut-PA*	Human *NPMc+*-cytoplasmic mutant; Myeloid	Normal hematopoietic complement	
*mRNA: NPMc+*	human Cytoplasmic *NPMc*+ mutant; global, transient	Embryonic increase of myeloid lineage	Enhanced myeloid bias in *p53* mutant line, increased apoptosis dependent on p53 (Bolli et al., 2010)
*spi-1: CREB-EGFP*	*creb*; Myeloid	Incidence 79%, latency 9–14 months; ∼66% monocytic leukemia	Similar expression profile to patients, identified 20 shared *creb* targets, blocks myeloid differentiation through *c/ebpδ*, biases monocytic subtype (Tregnago et al., 2016)
*fli1:GAL4-FF; UAS-GFP-HRAS^G12V^*	Human *HRAS^G12V^* mutant; Endothelial (hemogenic)	Myelo-erythroid proliferative disorder, expansion of CHT and myeloid progenitors	Caused by downregulation of Notch, can be rescued with Notch ICD expression (Alghisi et al., 2013)
*LDD731: CBL^H382T^*	*c-cbl^H382T^* mutant; global	Embryonic expansion of myeloid progenitors, lethal at 14–15 dpf	Increase in progenitors does not correspond with differentiation block, dependent on flt3 (Peng et al., 2015)
*irf8Δ57/Δ57*	*irf8* knockout; global	Embryonic myeloid expansion, decreased lymphoid, survive to maturity.	*mertk* signaling activated, required for myeloid neoplasia (Zhao et al., 2018)
**MDS**			
*tet-2^m/m^*	Enzymatically inactive *tet2*; global	Normal embryonic hematopoiesis, MDS at ∼24 months, myeloid progenitor dysplasia and anemia	Decreased 5hmC only in kidney marrow, redundancy of tet family in other tissues (Gjini et al., 2015)
*pu.1^G242D^*	Human *pu.1^G242D^ (spi-1)* mutant; global	Embryonic myeloid (granulocyte) expansion, phenotypes resemble MDS by 18 months	Anti-proliferative drug cytrabine, but not apoptosis drug daunorubicin, reduces granulocyte expansion (Sun et al., 2013)
*C-myb^hyper^*	Hyperactive *c-myb*; global	Embryonic myeloid (granulocyte) expansion, phenotypes resemble MDS by 1 year	MDS can progress to AML and ALL, are transplantable, and respond to *c-myb* target drug flavopiridol (Liu et al., 2017)

### Acute Myeloid Leukemia (AML) and Myeloproliferative Neoplasms (MPN)

Many hematological malignancies are driven by oncogenic fusion genes created after chromosomal translocations and these fusions can often be expressed in animal models or cell lines to drive transformation and oncogenesis. Zhuravleva et al. were the first to do so with a myeloid malignancy in zebrafish by creating transgenic fish expressing the *MYST3/NCOA2* (*MOZ/TIF2*) fusion product under the *spi-1 (pu.1)* early myeloid promoter (Hsu et al., 2004) along with *EGFP* (Zhuravleva et al., 2008). This fusion protein is the result of the inv(8)(p11q13) chromosome abnormality found in human AML, and fuses two histone acetyltransferases (HATs). A small number of F_0_ fish (1.1%) expressing the transgene developed AML after 14–26 months, characterized by expansion of myeloid blast cells and invasion of the kidney. This low incidence and long latency suggest that secondary mutations may be necessary to induce transformation.

Another model using the *NUP98-HOXA9 (NHA9)* fusion gene [t(7;11)(p15;p15)] was developed by Forrester et al. (2011) with an spi-1 promoter driving conditional expression of either *EGFP* or the transgene after heat shock by the *hsp70-Cre* line. This oncogenic fusion product is associated with poor prognosis in AML and chronic myeloid leukemia (CML) (Gough et al., 2011). Following heat shock at 24 hpf, *NUP98-HOXA9;Cre* embryos had perturbed hematopoiesis promoting myeloid fates, and also showed reduced apoptosis and cell cycle arrest in response to irradiation, correlating with increased levels of *bcl2*. 23% of *NUP98-HOXA9;Cre* fish developed myeloid tumors with a latency of 19–23 months. These tumors closely resembled the pathology of the polyclonal MPN found in *NUP98-HOXA9*-transgenic mice (Kroon et al., 2001). Further investigation into the model uncovered an increase in HSCs, as well as a dependency on *meis1*, the prostaglandin/cyclooxygenase pathway, and genome hypermethylation via *dnmt1* for the fusion gene’s oncogenic potential (Deveau et al., 2015). This dependency could be exploited through treatment with DNMT or COX inhibitors, or sub-therapeutic doses of either in combination with HDAC inhibitors. This study both revealed mechanistic details of the NHA9 oncogene and demonstrated the potential of zebrafish leukemia models in identification of new treatment combinations.

Because most leukemia oncogenes produce early detectable effects on hematopoiesis, along with the inherent advantages of the zebrafish model, it is possible to develop drug screens in preleukemic embryonic models. One such model was developed by Yeh et al. (2008) using the *AML1(RUNX1)-ETO* fusion oncogene under the heat shock responsive *hsp-70* promoter. After heat shock, embryos accumulated non-circulating immature blast cells, with disruption of definitive hematopoiesis via loss of *runx1* and *cmyb* expression, loss of *gata1-*expressing erythroid cells, and were ultimately driven to a myeloid-granulocytic fate. These effects were all downstream of *AML1-ETO*’*s* suppression of *scl*, and could be reversed with *scl* overexpression. The transcriptional signature of *AML1-ETO*-expressing embryos closely paralleled that of human AML. Using the perturbation of embryonic hematopoiesis and the AML transcriptional signature as a readout of *AML1-ETO* oncogenic activity, they were able to develop a chemical screen for inhibitors that can rescue *AML1-ETO’s* effects (Yeh et al., 2009). This screen identified the COX and β-catenin pathways as vital to the function of *AML1-ETO*.

*TEL(ETV6)-JAK2* fusion genes have been identified in both ALL and atypical chronic myelogenous leukemia (aCML), with slightly different translocations driving each, t(9;12)(p24;p13) and t(9;15;12)(p24;q15;p13), respectively (Peeters et al., 1997). Onnebo et al. (2012) created transgenic zebrafish lines expressing these different fusions under *CMV* or *spi-1* promoters to better understand how they drive oncogenesis distinctly. These lines differ from other fusion gene transgenic lines in that the fusion proteins were generated from the zebrafish *tel* and *jak2a* genes combined to mimic two different human translocations found in T-ALL and aCML. Overall, the different genes behaved true to form, with the T-ALL fusion gene disrupting embryonic lymphopoiesis and the aCML fusion gene disrupting myelopoiesis similar to an MPN, driven by either *CMV* or *spi-1*. They were also able to demonstrate subtle differences in activity, with the T-ALL fusion gene showing greater enzymatic activity, but reduced downstream STAT activation and decreased sensitivity to JAK2 inhibition.

Although Zhuravleva et al. were the first to claim production of AML in a zebrafish model, the first myeloid malignancy was created in the Zon lab. Le et al. (2007) generated β*-actin: LGL-KRAS*^G12D^*; hsp70-Cre* zebrafish, with conditional global expression of an oncogenic KRAS inducible by heat shock. This model produced a variety of tumors following heat shock, including rhabdomyosarcoma, myeloproliferative neoplasm, intestinal hyperplasia, and malignant peripheral nerve sheath tumor. Although the heat-shocked fish had juvenile lethality, they discovered that the non-heat shocked adult fish developed MPN, likely due to the well-known “leakiness” of heat-shock promoters. The MPN-affected fish displayed classic disease characteristics such as expansion of myeloid progenitors, invasion of the marrow (kidney), and depletion of erythroid cells. Interestingly, these MPN cells could engraft after primary transplantation into irradiated recipients, but were unable to engraft after secondary transplantation, suggesting that they lack self-renewal capabilities. Another model utilizing an oncogenic *RAS* mutation was developed by Alghisi et al. (2013) inducing expression in the hemogenic endothelium prior to hematopoietic emergence. This *fli1:GAL4-FF; UAS-GFP-HRAS*^G12V^ line developed an MPN characterized by prominent expansion of the CHT, increased number of immature hematopoietic cells, and a block of myeloid differentiation in the kidney marrow. The Notch pathway was significantly downregulated and overexpression of the active NICD rescued the MPN phenotypes. They used this model to identify candidate genes both downregulated by Notch and upregulated by RAS that could be involved myeloid oncogenesis.

Similar to the connection of *c-Myc* to T-ALL, *n-Myc* is frequently upregulated in AML and is a poor prognostic marker. Shen et al. (2013) created a heat shock responsive zebrafish line expressing murine *n-Myc*, *MYCN:HSE:EGFP*, that simultaneously drives expression of *EGFP*. Following heat shock, *n-Myc* overexpression promoted immature myeloid blast cell expansion and enhanced the repopulating activity of myeloid cells. *N-Myc* enhanced primitive hematopoiesis by upregulating *scl* and *lmo2* expression and promoted myelopoiesis by inhibiting *gata1* expression and inducing *spi1* and *mpo* expression. Many major cancer pathways were upregulated, such as cell cycle, glycolysis/gluconeogenesis, MAPK/Ras, and p53-mediated apoptosis. In contrast, mismatch repair and transforming growth factor β (TGFβ) signaling were downregulated. Overall, the model faithfully recapitulates AML phenotypes with high incidence (∼75%) and rapid onset (∼60 dpf).

Internal tandem duplications of the receptor tyrosine kinase *FLT3 (FLT3-ITD*) is a common mutation in AML and associated with poor prognosis and increased risk of relapse (Takahashi, 2011; Hou et al., 2013). It frequently coincides with mutations to the nucleophosmin *NPM1* that restrict it to the cytoplasm (*NPMc+*). Lu et al. (2016) sought to investigate the interaction of these two mutations in AML by making transgenic lines expressing each under the myeloid spi1 promoter, *spi1:FLT3-ITD-2A-EGFP and spi1:NPM1-Mut-PA.* The *FLT3-ITD* mutant fish alone developed moderate myeloid hyperplasia at 6 months and some of these progressed to leukemia at 9 months. *NPMc+* mutants had grossly normal hematopoietic composition. However, double mutants for both *FLT3-ITD* and *NPMc+* progressed to leukemia by 6 months, demonstrating their synergistic effect in driving AML. In a different model using *NPMc+* mRNA embryonic microinjections, Bolli et al. (2010) saw an increase in *spi1^+^* early myeloid progenitors, with a more pronounced effect in a *p53* mutant line. *NPMc+* expression resulted in increased erythromyeloid progenitors in the posterior blood island and *c-myb/cd41*^+^ cells in the ventral wall of the aorta. They suggest these results may be relevant to human *NPMc+* AML, where a multilineage expression pattern implies transformation of a multipotent HSPC.

Using a large-scale ENU mutagenesis screen, Peng et al. (2015) identified a line with a significant increase in HSPCs in hematopoietic organs, designated *LDD731:CBL*^H382T^. They determined the causal mutation was in the *c-cbl* gene, which is found frequently mutated in human MPN and acute leukemias and acts as a tumor suppressor by depressing growth factor and cytokine signals. The mutation was homozygous lethal at ∼15 dpf and led to an expansion of the myeloid/erythroid lineages in definitive hematopoiesis. *Flt3* was necessary for this expansion, consistent with that observed in both mice and humans, suggesting *flt3* signaling promotes HSPC proliferation and is regulated by *c-cbl*.

cAMP response element binding protein (*CREB*) is another frequently overexpressed gene in AML, however, it is unclear whether overactivation alone is sufficient to induce leukemia. Tregnago et al. (2016) generated a zebrafish model overexpressing *creb* with the *spi1* myeloid promoter, which resulted in a disruption of myelopoiesis in 79% of adult fish with 66% progressing to a monocytic leukemia (latency 9–14 months) mirroring the human counterpart. These fish showed a transcriptional signature with 20 differentially expressed genes in common with pediatric AML, including the CCAAT-enhancer-binding protein-δ (*c/ebp*δ). Increased *c/ebp*δ expression impaired myeloid differentiation which could be reversed through silencing of the *creb*-*c/ebp*δ axis. Identification of this *creb-c/ebp*δ axis in zebrafish AML led Tregnago et al to classify *C/EBP*δ expression as a new pediatric AML subgroup after validation in publicly available patient databases.

To study the role of interferon regulatory factor 8 (IRF8) in the pathogenesis of myeloid neoplasia, Zhao et al. (2018) created a missense mutation, *irf8*^Δ57^ that acted as a functional knockout. IRF8 is a critical transcription regulator for myeloid lineage commitment and closely tied to myeloid leukemia. *irf8* mutants quickly developed MPN with expansion of myeloid precursors, which recurred after transplantation, and invasion of kidney marrow. Myeloid expansion was caused by both increased proliferation and decreased apoptosis. *mertk* expression was increased in irf8 mutants leading to hyperactivation of the ERK pathway. Transgenic *mertk* overexpression recapitulated the myeloid neoplasia and knockdown of *mertk* rescued irf8 mutant myeloid expansion. These results support *mertk* signaling as critical in the *irf8*-mediated regulation of myeloid proliferation and survival.

### Myelodysplastic Syndrome

Myelodysplastic syndromes (MDS) are a group of diseases characterized by aberrant hematopoietic differentiation leading to cytopenias and increased blasts, and often splenomegaly and cytogenetic abnormalities (Gangat et al., 2016). Approximately 30% of MDS patients will eventually progress to AML or other leukemias, which are frequently more resistant to conventional therapies.

Somatic loss-of-function mutations of the 10–11 translocation 2 gene *TET2* are frequently observed in patients with MDS. *TET2* encodes a DNA methylcytosine oxidase that converts 5-methylcytosine (5 mC) to 5-hydroxymethylcytosine (5 hmC) to initiate the demethylation (and activation) of DNA. Gjini et al. (2015) created an enzymatically inert *tet2* mutant zebrafish line through genome-editing technology. These fish had normal embryonic hematopoiesis, but developed progressive clonal myelodysplasia as they aged, eventually progressing to MDS by 24 months, with myeloid progenitor cell dysplasia and anemia. Decreased levels of 5 hmC were present in hematopoietic cells of the kidney marrow but not in other cell types, likely a result of compensation in non-hematopoietic tissues by other Tet family members.

The *c-myb* transcription factor is vital to hematopoietic proliferation and differentiation, and is closely associated with an array of hematological disorders. Liu et al. (2017) sought to better define its pathogenic role through characterization of a zebrafish model expressing a GFP-tagged *c-myb* mutant with increased activity, *c-myb*^hyper^. This hyperactive c-myb resulted in the dysregulation of cell cycle genes and subsequent proliferation of hematopoietic progenitor cells. Abnormal granulocyte expansion began embryonically and was maintained through adulthood, ultimately resulting in MDS. A small number of *c-myb*^hyper^ fish developed AML or ALL and treatment with *c-myb* target drug flavopiridol relieved the MDS-like symptoms in both embryos and adult fish.

In addition to its previously discussed role in early myeloid progenitors, *spi1 (pu.1)* is an Ets-family transcription factor important in leukemogenesis. It is frequently impaired in AML either through decreased expression or loss-of-function mutations (Mueller et al., 2002; Dakic et al., 2007). Sun et al. (2013) used the Targeting Induced Local Lesions IN Genomes (TILLING) approach to create a hypomorphic *spi1* mutant allele, dubbed *pu.1*^G242D^. These fish have expanded myelopoiesis by 3 dpf, with increased immature granulocytes within the CHT. By 18 months, immature myeloid cells were increased at the expense of the lymphoid population in both the kidney marrow and peripheral blood, consistent with an MDS or AML-like disorder. The antiproliferative drug cytarabine was able to relieve the myeloid expansion, while apoptosis-inducing daunorubicin could not. This may indicate that spi1-associated neoplasms are more susceptible to drugs limiting their proliferation.

## Future Directions and Emerging Methods

The discoveries described in this review open numerous avenues for further research. Many of these models require further characterization and could uncover important pathways in leukemia initiation and progression. Drug screens utilizing these models can teach us much about the resistance and response to different therapies depending on the specific genetic drivers of the leukemia. The advent of effective CRISPR-Cas9 protocols has allowed for a rapid advancement in the creation of knockout and transgenic zebrafish investigating various genetic pathways and oncogene fusion products tied to human leukemia. This development will only continue to expand in scope, as ongoing research within the zebrafish field continues to uncover more genes and pathways associated with leukemia, as well as new discoveries made in the clinic that are converted into zebrafish models for further characterization.

One major emerging avenue of research is the identification and characterization of LSCs within hematopoietic malignancies. Cancer stem cells (CSCs), defined by their ability to regrow a tumor from a single cell, are implicated as the cause of cancer evolution, resistance to therapy, and relapse after therapy (Adorno-Cruz et al., 2015). Increased tumor heterogeneity and CSCs have been associated with resistance and relapse for many tumor types, including AML and ALL (Mullighan et al., 2008; Anderson et al., 2011; Notta et al., 2011; Ding et al., 2012). Identification and isolation of these cells is difficult because of a lack of defined surface markers, but there are some promising methods being developed. The side population assay has long been used to isolate normal tissue stem cells by exploiting their ability to export Hoechst dye (Goodell et al., 1996), and has more recently been shown to enrich for CSCs in many cancers (Hu et al., 2010; Britton et al., 2011; Richard et al., 2013). Side populations have also been defined in zebrafish hematopoietic cells and leukemia (Kobayashi et al., 2008; Pruitt et al., 2017), making it possible for LSCs to be further studied in zebrafishleukemia models. Another similar protocol of enriching for stem cell activity is through the Aldefluor assay, which utilizes the increased aldehyde dehydrogenase (ALDH) activity common in stem cells to produce increased fluorescence from the aldefluor reagent (Storms et al., 1999; Ma et al., 2010). This assay can also be combined with the side population assay to isolate an even greater enrichment of stem cells (Pearce and Bonnet, 2007; Pierre-Louis et al., 2009). Genetic and functional characterization of the combined ALDH^*bright*^ and side population in zebrafish leukemia models could uncover significant contributors to leukemia resistance and relapse.

Another potential method for defining LSCs within a tumor is through single cell sequencing. Single-cell RNA sequencing techniques are capable of discerning expression profiles of each cell within a population, allowing small subpopulations like LSCs to be characterized within a tumor (Zhang et al., 2016). Multiple microfluidic systems have been developed to produce single-cell expression data, with the Fluidigm system already used by Moore et al. (2016) in a zebrafish T-ALL model to identify a small population with reactivated expression of putative stem cell genes. The DropSeq system is an alternative with much higher throughput that could potentially identify very small subpopulations in tumors with lower LSC frequencies (Macosko et al., 2015). Both systems allow for characterization of the expression profiles of LSCs in the various leukemia models, which opens up exciting possibilities in discovering what drives the LSC subpopulation and their unique functions within the leukemia.

## Author Contributions

JB wrote the entirety of the review, with significant input from JdJ in the initial outline and also comments and revisions of the final version.

## Conflict of Interest Statement

The authors declare that the research was conducted in the absence of any commercial or financial relationships that could be construed as a potential conflict of interest.
